# Barriers and enablers to emergency obstetric and newborn care services use in Wolaita Zone, Southern Ethiopia: a qualitative case study

**DOI:** 10.1186/s12889-022-14504-y

**Published:** 2022-11-16

**Authors:** Mihiretu Alemayehu, Bereket Yakob, Nelisiwe Khuzwayo

**Affiliations:** 1 School of Public Health, College of Health Sciences and Medicine, Wolaita Sodo University, Wolaita Sodo, Ethiopia; 2 School of Nursing and Public Health, Discipline of Public Health, University of KwaZulu-Natal, Durban, South Africa; 3 School of Population and Public Health, the University of British Columbia, Vancouver, BC Canada

**Keywords:** Obstetric service, Neonatal service, Emergency obstetric service, Maternal mortality, Maternal health, Maternal and child health

## Abstract

**Background:**

Globally, 11.4 million untreated obstetric complications did not receive Emergency Obstetric and Newborn Care (EmONC) services yearly, with the highest burden in low and middle-income countries. Half of the Ethiopian women with obstetric complications did not receive EmONC services. However, essential aspects of the problem have not been assessed in depth. This study, therefore, explored the various aspects of barriers and enablers to women’s EmONC services utilization in southern Ethiopia.

**Methodology:**

A qualitative case study research design was used in nine districts of the Wolaita Zone. A total of 37 study participants were selected using a purposive stratified sampling technique and interviewed till data saturation. Twenty-two key informant interviews were conducted among front-line EmONC service providers, managers, community leaders, and traditional birth attendants (TBAs). Individual in-depth interviews were conducted among 15 women with obstetric complications. The trustworthiness of the research was assured by establishing credibility, transferability, conformability, and dependability. NVivo 12 was used to assist with the thematic data analysis.

**Result:**

Five themes emerged from the analysis: service users’ perception and experience (knowledge, perceived quality, reputation, respectful care, and gender); community-related factors (misconceptions, traditional practices, family and peer influence, and traditional birth attendants’ role); access and availability of services (infrastructure and transportation); healthcare financing (drugs and supplies, out-of-pocket expenses, and fee exemption); and health facility-related factors (competency, referral system, waiting time, and leadership).

**Conclusion:**

Many women and their newborns in the study area suffered severe and life-threatening complications because of the non-utilization or delayed utilization of EmONC services. A key policy priority should be given to enhancing women’s awareness, eliminating misconceptions, improving women’s autonomy, and ensuring traditional practices’ role in EmONC service utilization. Community awareness interventions are required to enhance service uptake. Furthermore, the health systems must emphasize improving the quality of care, inequitable distribution of EmONC facilities, and essential drugs. The financial constraints need to be addressed to motivate women from low socioeconomic status. Furthermore, intersectoral collaboration is required to maintain a legal framework to control and prohibit home deliveries and empower women.

**Supplementary Information:**

The online version contains supplementary material available at 10.1186/s12889-022-14504-y.

## Introduction

Globally, an estimated 295,000 women die due to pregnancy and childbirth-related complications, with the highest burden in low and middle-income countries (LMICs) [1]. Recent evidence shows that Ethiopia still faces a considerable burden of maternal and newborn morbidity and mortality [2] despite the substantial improvements in access to and availability of EmONC facilities [3].

Emergency obstetric and neonatal care (EmONC) (the care given during pregnancy, childbirth, and the postpartum period when severe and life-threatening complications occur) is believed to avert the majority of deaths if all mothers get quality health care [4]. Evidence shows that ‘three delays’ contribute to maternal and neonatal deaths in developing countries [5]. The lengths of the delays in (a) the decision to access care, (b) the identification of and transport to a medical facility, and (c) the receipt of adequate and appropriate treatment have a direct relation to the outcome of obstetric emergency [6, 7]. This suggests that for effective maternal and newborn health strategies, accessible, need-based, and quality EmONC service is essential [4].

Globally, more than half of women with obstetric complications do not receive EmONC services, with a significant disparity between low- (21%), middle- (32%), and high-income countries (99%) [8]. This disparity corresponds to an annual 11.4 million untreated complications and 951 million women without access to the EmONC service [8]. The difference is visible within Sub-Saharan African countries ranging from 9.9 to 65.1% met-need for EmONC services [9–13], with inequitable service distribution across the study areas. Despite substantial investments in the scaling-up of health services in recent years, half of ‘Ethiopia’s women with obstetric problems do not receive EmONC services [14].

Health systems deliver optimum health services when those in need access services and receive high-quality care [15]. In low-income countries, health systems face several challenges [16], and women needing EmONC services often have difficulty accessing and utilizing them [17]. Though several studies assessed the EmONC service utilization, many assessed it quantitatively, and limited evidence exists regarding women’s contextual challenges when seeking care from health facilities. Accordingly, essential aspects of the problem, such as interpersonal, traditional, community, and health system-related barriers and enablers to EmONC service, have not been assessed in-depth, particularly in Ethiopia. This study, therefore, qualitatively explored the various aspects of barriers and enablers to women’s EmONC services utilization in southern Ethiopia.

## Methods

### Study setting

The study was conducted in the Wolaita Zone, southern Ethiopia, located 330 km south of Addis Ababa, the capital of Ethiopia. This Zone is projected to have more than 2.6 million population in 2020 [18]. The Zone has ten hospitals (including one comprehensive specialized hospital), 70 health centers, and 326 health posts [19], providing services to the people dwelling in the Zone and other neighboring zones.

### Study design

The study used a qualitative case study research design [20] and explored the barriers and enablers of EmONC services by gathering data from multiple sources. Case study research helps to investigate the circumstances, such as ‘how’ or ‘why’ some phenomenon works or does not work in a particular context [20]. The method was relevant for the current study since the study required an extensive and in-depth description of the phenomenon (EmONC service utilization-related barriers and enablers) and how it was influenced by the context within which it was situated [21].

### Boundaries of the study

This study’s case is EmONC services. The activity boundary of the case is service utilization. It is further bounded conceptually - barriers and enablers of EmONC service use; geographically -Wolaita Zone, and temporally -study period of April 01 – August 31, 2020. The actor/informant boundary of the study was- women with obstetric emergencies, service providers (health care professionals and traditional birth attendants), and facility and community leaders. As the causes for potential perceived success (utilization of health service) are numerous and diverse, theories such as the health belief model [22], attribution theory [23], the WHO health system framework [24], and the three delays model [5] were reviewed and assisted with developing a framework to guide the study and identify the unit of analysis. Accordingly, five units of analysis were identified: service users’ perceptions and experiences, community-related factors, access and availability of services, healthcare financing, and health facility-related factors.

### Sampling and participant selection

Thirty-seven participants (22 key informant interviews and 15 individual in-depth interviews) participated in the qualitative inquiry. Potential study participants were selected through the *purposive stratified sampling technique* [25], using the *maximum variation sampling* method. Accordingly, the study participants were selected from various perspectives, such as service providers, service users, and other individuals, including traditional birth attendants and women development army leaders, who can provide rich and tick perspectives and experiences of EmONC services utilization. Besides, to increase variation in the sample, study participants were selected after stratifying them into rural and urban sites, types of obstetric complications, and pregnancy outcomes. The number of participants was determined by the principle of theoretical saturation of data***.*** Potential participants were selected based on their attributes to the research question.

Seven front-line EmONC service providers and seven managers/heads were recruited from health facilities and district and zonal health offices based on their experience and knowledge of EmONC services. Nine women from health facilities (one from each facility) and six from the community (homes) who had obstetric emergencies were selected to obtain rich information on barriers and facilitators of EmONC service use. Although the facility-based recruitment addressed women’s experience with EmONC service utilization, it mainly focused on the challenges of receiving quality EmONC services. In contrast, women from the community were chosen to explore barriers to EmONC service utilization. Therefore, they were selected based on the criteria of not utilizing EmONC facilities, despite the experience of having obstetric emergencies (complications).

Furthermore, the study recruited community leaders (kebele/village leaders and health development army leaders) to explore the perspectives of the community regarding EmONC services delivery and community-related barriers and facilitators of EmONC service utilization. Therefore, the interview guide focused on the socio-cultural factors related to the non-utilization of EmONC services and the community’s perception of quality of care. Besides, traditional birth attendants (TBAs) were also interviewed to unveil why women failed to seek care from health facilities. This study, therefore, collected data from multiple sources to holistically explore the complex situation of EmONC service use-related barriers and facilitators.

### Data collection

This study used semi-structured interview guides for key-informant interviews (KIIs) and individual in-depth interviews (IDIs). The principal investigator developed the interview guides and was assured by the co-investigators to explore the barriers and enablers of using the EmONC service. Five interview guides (Additional file 1) were used and conducted in the local languages, Wolaita Dona or Amharic (where applicable), by the principal investigator (male) along with a female research assistant (with a Master’s degree in Public Health and experience in qualitative research) using a tape recording of audios and field notes.

Confidentiality and privacy of the participants and information were maintained by conducting the interviews in offices, staff rooms (quiet places in participants’ working units), and homes at their convenient times. The interviews were initiated after clearly informing the aim of the study and receiving permission (consent). The interviews began with general questions to elicit interest. Besides, efforts were made to establish rapport between the interviewer and respondents. The interviews lasted 30 to 60 minutes.

The health facility leaders and service providers were interviewed at their convenient time in a separate room in their respective facilities. The content of the interview guide mainly focused on the challenges related to EmONC service provision and quality of care. Women selected from health facilities were interviewed in a separate room in the respective facility where no one was allowed to enter so that they confidently interacted with the interviewer and responded freely. The interviews of those women chosen from the community were conducted in their homes conveniently to ensure they behaved naturally without interruption. At convenient times, the community leaders’ interviews took place in their respective kebele offices (peasant association office, the smallest local administrative structure in Ethiopia). Besides, traditional birth attendants (TBAs) were also interviewed at their homes without interruption.

### Data analysis

The overall compositional structure of the study was guided by the *linear-analytic structure* approach [20], which started with the research problem and ended up with a conclusion and implications for the problem studied. The quality of the study was guided by the COREQ (Consolidated Criteria for Reporting Qualitative Research) checklist (Additional file 2) [26].

Data analysis started while collecting data to identify emerging themes for the consecutive interviews. The principal investigator transcribed the data verbatim and translated it into English. The transcription was rechecked with the original data to confirm consistency. The data were read and re-read by the investigators. NVIVO (QSR International Pty Ltd. Version 12) qualitative data analysis software was used for data coding and analysis. Codes were identified and grouped into themes. The principal investigator coded the data. The three investigators organized the data to analyze the results thematically in analytical categories based on the research aim and ideas arising during the data collection. The study adopted Braun and Clarke’s six steps for thematic analysis, which include*(a)* familiarization *with data, (b) generation of codes, (c) searching, (d) revision, (e) definition and naming themes, and finally (f) producing the report* [25].

This study applied an *integrated coding approach* that relied on theoretical propositions and analyzed the data from the ground up [20]. This analysis strategy assisted the study in defining codes deductively and inductively. Some codes were defined deductively (derived from literature) before starting analysis, and some were defined inductively (emerged from the data) not to miss relevant data (codes) [20, 27, 28]. Hence, a *logic model* case study analytic technique was used for the analysis since it guided in matching empirically observed events identified through the inductive coding to theoretically predicted events of the deductive coding [20].

### Trustworthiness of the study

Initially, the investigators prepared the study protocol, including the aim, study design, data collection, analysis, and reporting. Based on the protocol’s activity plan, the collected data, procedures, and study steps were well-documented and stored confidentially and privately. The study tool was translated into the local language for the interview. The principal investigator and research assistant were fluent speakers of the local language and familiar with the study community. Besides, all members of the research team (two with a Ph.D. in Public Health and one Ph.D. fellow) with prior experience with qualitative research and adequate academic background conducted the data collection. This helped the study maximize the *credibility* of its findings.

Data were collected from multiple sources, including women who had various types of obstetric emergencies, health care providers, health facility managers, traditional birth attendants, and community leaders. Hence, the themes were built by converging data from multiple sources (perspectives of participants), enhancing the ‘study’s *credibility*. There were regular discussions and briefings between investigators that assisted in minimizing mistakes during analysis. This enhanced the *confirmability* of the study. The investigators, therefore, understood emerging codes and categories in the same way. Repeated interviews of similar settings were done till theoretical saturation of data was attained, which enhanced the *transferability/dependability* of the study. As a result, the study revealed a rich and detailed description of the EmONC utilization-related challenges. The final report was presented to discuss the major findings. Then, the overall report was examined by maternal and child health scholars in the study area, and experts (in the field of study) were consulted.

### Ethical considerations

The study was approved by the Biomedical Research Ethics Committee of the University of KwaZulu-Natal, South Africa, and the Institutional Review Board of Wolaita Sodo University, Ethiopia. After receiving permission from the Wolaita Zone Health Department, the ‘study’s data collection process was initiated. Furthermore, respondents received informed consent after explaining the ‘study’s goal and communicating the privacy and confidentiality of their responses.

## Result

### Characteristics of study participants

The participants’ characteristics are shown in Table 1. The mean age of participants was 31.2 years. Most of them were females, government employees, and in the age range of 25-34 years old. All participants gave consent to participate in the study.

**Table 1:** Sociodemographic characteristics of participants, EmONC service utilization-related challenges, Wolaita Zone, Southern Ethiopia

Characteristics		Number
Age	< 25	5
	25-34	22
	>= 35	10
Sex	Male	12
	Female	25
Education	Cannot read and write	10
	Primary (Grade 1-8)	5
	Secondary (9-12)	4
	College/University	18
Occupation	Housewife	4
	Government employee	19
	Other^a^	14

^a^Farmer, merchant, student, TBA, daily labor

**Fig. 1:**
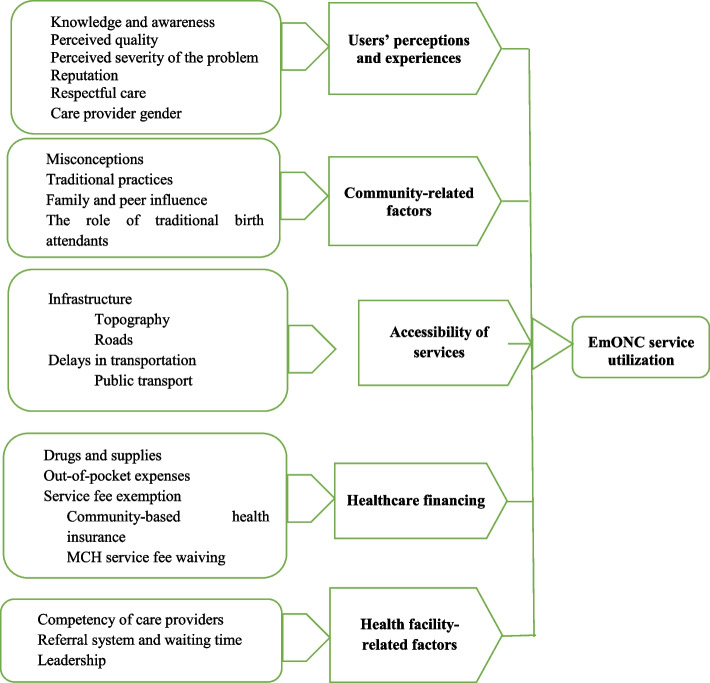
Barriers and enablers of EmONC service utilization in Wolaita Zone, Southern Ethiopia, 2022

The barriers and enablers of EmONC service utilization were analyzed in five themes: 1) service users’ perception and experiences, 2) community-related factors, 3) access and availability of services, 4) healthcare financing, and 5) health facility-related factors. (Fig. 1).

#### Service users’ perceptions and experiences with EmONC services

##### Knowledge and awareness of EmONC service availability

Many women had appeared unaware of the availability of EmONC services and their benefits. Some women delivered their babies at home, and others used traditional birth attendants. Most women were exposed to the EmONC services for obstetric complications when they were first admitted to the ward. They explained that they were not informed about the services or were unwilling to accept the advice until they faced the current obstetric emergencies. Unlike most women who delivered at home or ‘TBA’s home, women who visited health facilities for obstetric emergencies explained that they valued the service as lifesaving.

For instance, a 30 - year old woman who lost her baby because of the delay in seeking care from EmONC facilities explained the impact of lack of awareness on her health and wellbeing as follows:

*‘I did not know the availability of such services given to mothers with pregnancy-related emergencies. This is my third delivery at home. I have no information about the benefit of giving birth at a health center* (IDI, WC#1, a 30-year-old woman with stillbirth)

*‘Problems such as excessive bleeding … could be stopped and our lives could be saved if we visit health facility sooner .... Many women with similar conditions like me need an operation [cesarean section] and save their babies.’* (IDI, WF#2, 26-year-old woman with pregnancy-induced hypertension)

On the contrary, although some women have access to health services, they fail to use them, and some use the services later in their pregnancy after adverse health outcomes. Some women explained that the lack of awareness negatively affected their health and wellbeing.

*‘We frequently came to Sodo town for our livelihood and we knew a big hospital there. But, we thought labor-related health problems could be managed at home rather than going to the hospital’. (IDI, WF#5, a 20-year-old woman with postpartum hemorrhage)*

##### Perception of the quality of care

Women perceived that the quality of EmONC services was ‘poor.’ This valuation was substantiated by their experiences in health facilities with various obstetric complications. For instance, many women who visited EmONC health facilities said that they were severely ill, their babies died, and/or had heard/seen their neighbors or their babies die due to preventable obstetric complications. The women mentioned that poor quality caused the women’s deaths and other adverse health outcomes. They explained that their perceptions of the quality of care provoked them to refrain from visiting health facilities for obstetric emergencies.

*‘We are not interested in going there [health facility] because their service is poor.*’(IDI, WF#1, a 30-year-old woman with prolonged labor)

##### Reputation and respectful care

Respondents explained that the health facilities’ reputation was a motivating factor for women’s EmONC service use. Respondents mentioned that the community had its own informal *‘criteria’* (mostly care providers- related factors and availability of drugs) for health facility preference to visit. They emphasized that the presence of a health facility with passionate care providers in their locality was an encouraging factor for women’s EmONC service use and vice versa.

*‘ …*. *I prefer the Wamura health center though Shamba health center is nearer to my home. I preferred Wamura because health professionals are always ready to take care of us … ’* (IDI, WF#6, a 26-year-old woman with postpartum hemorrhage)

According to IDIs with the women, experiencing disrespect and abuse discouraged women from using EmONC services in the study area. They mentioned that some care providers *slapped and shouted* at them. Furthermore, some women reported being neglected or left unattended for days and nights by care providers. They also said some women with life-threatening conditions and severe labor pain are left unattended by care providers.

*‘I shouted at one care provider because no one observed me the whole night though I was suffering from the wound pain. He told me to calm down, but I continued to be emotional. Then, he slapped me and told me to stay calm and wait till they got time to observe me. It is too rude to do so for a mother like me who lost her baby and suffered more on operation [cesarean section]’ (IDI, WF#3, a 26-year-old woman with stillbirth)*

*‘Health professionals are not punctual when a mother arrives at the health center. … Women face disrespect and negligence from care providers. Some health care providers even insult them. So, women, their attendants, and families lose interest and refuse to revisit that facility.’* (KII, CL#4, a 27-year-old women’s development army leader)

Despite the care providers’ misconduct (that discouraged women from EmONC service use), humble, compassionate, and caring care providers’ existence was a facilitating factor for EmONC use.

*‘The care providers in the health facility immediately secured the IV line while asking me questions about labor duration and the steps we took. They rushed to help me … called the ambulance to refer me to the hospital. They were humble and caring. This is really motivating to visit again in the future … ’ (IDI, WF#5, 20-year-old woman with PPH)*

##### Care provider gender

Besides, care providers’ gender was also implicated in their perceptions of quality. Most of them said they preferred male care providers to females since they perceived those male providers were more passionate and understood their interests better. They reported that every woman would be interested in visiting health facilities if all the facilities hired such compassionate and respectful professionals.

*‘ … unlike the female care provider mistreating me, the male care provider treating the woman next to me in the delivery room was an excellent care provider. I observed his commitment and passion for helping the woman.’* (IDI, WF#6, 26-year-old woman with PPH)

#### Community-related factors

##### Misconceptions

Based on their previous home delivery experiences, some respondents perceived all pregnancies and labor as usual as in their previous deliveries. For instance, women who didn’t access medical care for obstetric emergencies perceived the complications as self-healing health problems.

*‘I was waiting for days for it [the bleeding] to stop by itself because, in our community, bleeding is perceived as normal during childbirth … so people around me were praying now and then.’* (IDI, WC#3, 23-year-old woman with PPH)

##### Traditional management of obstetric complications

Some respondents explained the impact of traditional management of obstetric complications and its contribution to home delivery and delayed arrival at the health facility. The community has various local terms for obstetric complications, such as ‘*xessayminttiis*’ (waist tightness) for obstructed/prolonged labor, ‘*sugettaa’* for neonatal distress, and traditional feeding habits like drinking a locally made drink composed of wormwood herb (*Artemisia absinthium*), milk, and milk products to decrease labor pain and prevent delivery-related complications*.* Those women who delivered at home also explained that the people around them (most from rural settings) prefer to get treatment at home than go to health facilities.

*‘ … whenever newborns become sick, our society traditionally perceives it as ‘sugettaa,’ and they massage the newborns’ abdomen.* (KII, CL#2, a 32-year-old women development army leader)

##### The role of traditional birth attendants

TBAs were found to be facilitators and barriers in the EmONC service utilization. They described their role, including making referrals to the health facility, as vital since they strive to reduce maternal deaths. All of them explained that they refer mothers when the complications worsen. On the contrary, the availability of TBAs in the community held back women’s EmONC service use. When women faced life-threatening obstetric complications, they thought that the traditional birth attendants (neighbors and family) would manage it. A 26-year-old woman with stillbirth after delayed arrival at EmONC facility explained the TBA’s role on her health and wellbeing as follows:

*‘I know my labor was not so bad, but the local birth attendants made my labor worst. … they were telling me that the baby would be born very soon since its head was visible. … and I also believed their words and wished to see my baby soon. However, I lost my baby after three-day long labor … hoping and believing their words.’* (IDI, WF#3, 26-year-old woman with stillbirth)

TBAs explained that they prepare local (traditional) medicine to manage obstetric complications. Most use a locally made medication to treat/manage obstructed/prolonged labor (which they call ‘waist tightness’). They also manage/treat hemorrhages (postpartum or antepartum) by exerting pressure over the mother’s lower abdomen or vagina till the bleeding stops. The procedure is time-consuming, so waiting a few hours is needed to see the effect. When the effect is not observed after hours (it might last the whole day and night), they decide to send the mother to a health facility.

*‘I prepare a mixture of drinks made from ‘natira’ [*local name for *Artemisia absinthium], butter, and yogurt, … and order her to drink it, which eases the labor and gives strength to the mother. I thoroughly massage the womb with my hands if she has waist tightness [prolonged/obstructed labor] or rotate the baby manually when she faces Marsha [breech presentation]. ... sometimes, the bleeding [antepartum hemorrhage] becomes difficult to stop … and the mother loses energy … In that case, I refer her to health facility … ’* (KII, TB#1, 50-year- old TBA)

The TBAs also perceived better quality care at health facilities than theirs. However, many women preferred to be served by TBAs mainly because of the poor approach of healthcare providers and the friendly approach of TBAs.

*‘ … Even I cut the cord by using a blade that I wash with boiled water … I know that the health facility has gloves, syringes, drugs … However, women prefer me because I am always available when they seek me. I also encourage and give hope to them’* (KII, TB#2, 48-year-old TBA)

##### Family and peer influence

The husband, family, neighbors, and others were found to have a significant role in the decision for EmONC service use. Many women, who failed to seek EmONC service, explained it as a *‘decision of others’* than executing their interest. Community leaders and women described that the close interaction with peers and neighbors resulted in believing and accepting their advice to stay at home, regardless of the complications’ severity. Besides, women also explained that arguing with aged women’s thoughts would affect their smooth interaction with the community. Hence, in some circumstances, they accept (unwillingly) to stay at home and call the traditional birth attendants.

*‘ … everyone around me, my husband, my neighbors, … encourage giving birth at home. … many women in my village gave birth at home.’* (IDI, WC#8, a 29-year-old woman with sepsis)

#### Accessibility of services

##### Infrastructure

The difficulty in accessing health facilities was also among the barriers to EMONC service utilization. Participants mentioned distance from the facility and the locality’s difficult, rugged topography as significant barriers to EmONC service use. The distance did not matter much in some cases, but the topography made it difficult to arrive at the facility on time.

*‘They decided to carry me to the health center … my home is just beneath [near to] mount Damota. ... so, the landscape was challenging even for the people carrying me. [Then] They changed their minds and decided to visit our village’s known [famous] traditional birth attendant. … we were taking a rest in between the footwalks. … finally, after a three-hour walk, we arrived at the traditional birth attendant’s home.’* (IDI, WC#9, a 22-year-old woman with stillbirth)

Some respondents described that though women had an interest and good perception of EmONC services, they could not get the service because of difficulties in the accessibility of facilities.

*‘ … after I faced a falling accident when fetching water from the river, a few minutes later, … I started to see blood coming out from my private part [genitals]. … the place is out of rich of transportation … so, I was waiting for it [remnants] to expel completely … ’* (IDI, WC#3, a 19-year-old woman with post-abortion complication)

##### Delays in transportation to the medical facility

In contrast to the distance and topography of the localities, respondents also explained that some facilities are located in relatively accessible places. However, the limited availability of public transport was the cause of the delay in EmONC service utilization. A 30-year-old woman with prolonged labor, who had a stillbirth, explained the challenges associated with the availability of public transport:

*‘ … my home is just on the roadside, but it was midnight so we could not get public transport. I arrived at the health facility in the morning, but … I was not lucky to hug my baby … ’ (IDI, WF#1, a 30-year-old woman with prolonged labor)*

#### Healthcare financing

##### Drugs and supplies

Respondents raised the availability of medical equipment and drugs as one of the major contributors to women’s preference for seeking EmONC services. An adequate supply of drugs and other facilities motivated women to seek care from health facilities. Nevertheless, they explained that the facilities frequently ran out of cheap medical supplies (such as gloves, syringes, and needles) and expensive drugs (such as ceftriaxone). Laboratory and radiology services frequently stopped functioning because they ran out of reagents/inputs. The care providers and facility leaders explained that these problems compromised the service provision, worsened complications, and discouraged women’s revisits.

*‘Recently, we are facing a frequent shortage of drugs. … we order patients to purchase also drugs, gloves, and syringes from private pharmacies. But women were unhappy, … frequently complaining and losing interest in seeking care.’* (KII, SP#3, 28-year-old midwife)

They explained that inadequate supply and lack of appropriate utilization of resources resulted in a shortage of supplies. Some care providers and facility leaders mentioned that the available supplies were dispensed and prescribed unreasonably. They explained that this further resulted in both care providers’ and users’ dissatisfaction with the service. The facility leaders also described that these shortages ultimately caused women’s loss of interest in seeking care from health facilities.

*‘We hear prescription and drug administration and control are weak at hospitals and health facilities. This caused patients’ dissatisfaction’* (KII, FL#6, 36-year-old program leader)

##### Out-of-pocket expenses

Fear of the unaffordability of food and transportation costs was also mentioned as a barrier to EmONC service use. Respondents explained that expenses related to long-distance travel and prolonged hospital stay negatively affected women’s health-seeking behavior. Respondents added that the extra economic burden exerted on women and their families because of purchasing expensive drugs from private drug stores did not allow many women to seek care from EmONC facilities.

*‘ … Women do not want to suffer from such challenges [mentioned above] in the health facility, so they refuse to go to health facilities.’* (KII, CL#1, 30-year-old kebele leader)

##### Service fee exemption

 Health office heads/managers, care providers, and community leaders mentioned the importance of community-based health insurance (CBHI) for improving the health-seeking behavior of the community in general. They explained it as a considerable asset mainly for the poor, who could not afford the ever-increasing medical cost and other equipment purchases. To EmONC services, the fee waiving for all maternal and child health services was mentioned as a facilitator of increased uptake of EmONC services.

*‘The community health insurance is gaining acceptance in our village. This supports their financial needs. … and governmental health facilities give maternal and child health services free. This eliminates the stress related to unplanned health care-cost expenditure. ‘*(KII, CL#1, 30-year-old kebele leader)

#### Health facility-related factors

##### Competency of care providers

Women preferred visiting health facilities with more qualified care providers and committed and well-experienced workers. Facility managers also emphasized that care providers’ training positively impacted women’s health-seeking behavior by enhancing their satisfaction with the service. They emphasized that women’s satisfaction was the main reason for revisiting the facilities for obstetric emergencies.

*‘We are recruiting care providers with minimal exposure and training in managing obstetric emergencies. But, I think this is not a major problem in hospitals because they have more qualified doctors. So, many women prefer to go to a distant hospital than get the service from our health center.’* (KII, FL#3, 30-year-old facility head)

##### Referral system and waiting time

Health care providers and health office heads/leaders highlighted that care providers with poor knowledge and skill usually refer women to higher-level facilities though the complication could be managed/treated there. Though women expected to get the service at their initial arrival at the health facility, they were further referred to other facilities (drug stores, laboratories, and tertiary facilities). Some women refrained from going to the referral facility, and others went unhappily.

*‘I was informed that the hospital gives mothers free of charge services. But, they referred me to a private facility for laboratory diagnosis. How can I afford 450 birrs [ETB]? … My fate is going home … ’* (IDI, WF#3, 26-year-old woman with stillbirth)

The unavailability of ambulances and drivers’ lack of passion were also barriers to timely EmONC service utilization. The challenge is more eminent during nightshifts. In some cases, the ambulances were available, but the drivers were not cooperative during midnights.

*‘ … we called the health extension worker, and she [the health extension worker] was repeatedly calling the ambulance driver, but he was not responding to her call the whole night. … ’ (IDI, WF#1, a 30-year-old woman with prolonged labor)*

The health facilities’ patient flow and waiting time were stated as a challenge for women’s EmONC service utilization. They explained that they were obligated to wait further within the facility to be diagnosed and treated. They explained that this further worsened their complications, despite the long travel duration’s impact.

*‘There was a huge queue, and since I had no referral letter, I was obligated to wait for hours. I don’t recommend this hospital to others because of the crowding and long waiting time … ’* (IDI, WF#4, 25-year-old woman with spontaneous abortion)

##### Leadership

Respondents also mentioned that the loose monitoring and evaluation contributed to the women’s lack of interest in utilizing EmONC services. They described that the lack of appropriate measures for misbehaving care providers was a triggering factor for the dissatisfaction of both service providers and users. They implicated it to the women’s lack of interest in visiting EmONC services. Besides, they added that the health facilities were not working hard to attract service users and make the facility a *patient-friendly* institution.

‘The delivery room lacks cleanliness and readiness for the next patient. Such issues drive back patients from revisiting our facility.’ (KII, FL#6, a 36-year-old program leader).

## Discussion

This study explored the barriers and facilitators of EmONC service utilization from multiple sources in the Wolaita Zone, southern Ethiopia. Though every woman has the right to attain the highest standard of health, including the right to dignity, and compassionate and respectful care [29], this study reported that many women and their newborns missed EmONC service utilization. Our findings show that various factors contributed to the women’s utilization/non-utilization of EmONC services during obstetric emergencies. Accordingly, the findings were merged under five themes: service users’ perception and experiences, community-related factors, access and availability of services, healthcare financing, and health facility-related factors.

### Service users’ perceptions and experiences

Despite the health system’s increased emphasis on enhancing the coverage and quality of obstetric services [30, 31], the current study reported that low awareness and negative perceptions of EmONC services impact their utilization. Previous safe home delivery or delivery with minimal complications emboldened women’s perception that *‘every pregnancy and home deliveries are safe.* Women also believed that obstetric complications subside by themselves. Evidence from Nigeria also showed that women misinterpreted the signs of obstetric complications [32] and that the perceived susceptibility and threat, and perceived severity [22] of obstetric complications affected women’s decision to seek care from EmONC facilities [33]. This problem is not limited to LMICs [34, 35]. Evidence shows that women’s expectations [34] and prior negative experiences with health services and care providers [35] negatively influenced maternity care utilization. The better perception of women who recently visited health facilities implied that behavior change interventions could substantially impact marginalized women’s behavior. Evidence from central Ethiopia also suggested that women’s health education had a noticeable effect on service utilization [36].

The perceived poor quality of the EmONC services in the study area, particularly fear of health professionals’ disrespect and abuse, were among the barriers to accessing EmONC services. In the current study, women’s experience of disrespectful care resulted in dissatisfaction with the obstetric care services. Besides, discrepancies between expectations and experienced care are also reported as important factors for women’s dissatisfaction with care [34, 35]. Since negligence, disrespect, and abuse were serious obstacles to maternal health services utilization [37], and they rid women of their right to have care with dignity, they must be taken seriously, and relevant authorities, such as local health administrations, take measures – including training care providers on respectful care and make frequent supervisions. Furthermore, efforts to improve the perceived quality of care need to balance women’s expectations of EmONC services and outperform their past experiences to satisfy them with the current services and enhance the utilization of such services in the future. This depicted that the quality of care is vital for EmONC service utilization.

### Community-related factors

The impact of some traditional practices was palpable in the current study, which tremendously affected women’s healthcare-seeking behavior. The TBAs’ role in referring women to health facilities was considered an enabling factor for EmONC service use in the current study. Nevertheless, it should be emphasized that recovery is less likely for women referred on the verge of adverse health outcomes. This is evident in the case of a study conducted in Tanzania, where many TBAs did not know the signs and symptoms of obstetric complications [38], which further contributes to delays in referral.

This study also identified that social networking influenced women’s decision to seek care from EmONC facilities, where the husbands, peers, relatives, and family were either sole or dominant decision-makers. The existing evidence has shown that the delay in the decision to access care is highly influenced by the decision-making power of women [5] and substantially contributes to maternal and neonatal death [6, 7, 39, 40].

### Access and availability of services

Though some women in the study area perceived EmONC services as a valuable component of maternal and child health services, they were not lucky to obtain them due to difficulties accessing the facilities. The main challenges they reported were distance, topography, and availability of public transport, which were also reported in other studies [32, 41]. These challenges are common in LMICs [7, 42–45] and could be addressed by improving the wise use of available infrastructures, such as ambulances, assigning a dedicated staff for the service, having a functional referral system, and soliciting logistics from nearby health facilities whenever possible. Besides, according to the Ethiopian health policy [30], EmONC services are waived of any fee, including ambulance services, which are crucial for the timely arrival of women with EmONC complications. However,in some instances, the drivers were accused of negligence and lack of cooperation. Besides, the health facilities’ poor resource management limited ambulance service in the current study. Hence, not only the lack of facilities or infrastructure but also poor resource management needs to be addressed to improve the availability and utilization of EmONC services.

### Healthcare financing

Though Ethiopia has nationally uniform guidelines for health centers and hospitals [30], the availability of medical equipment and essential drugs varied across facilities in the study area. This mainly compromised the quality of care and further discouraged the revisit of women for their obstetric emergencies. Besides, the extra economic burden (transportation and food cost) exerted on women from low socioeconomic classes did not allow many women to seek care from EmONC facilities. Similarly, minorities in high-income countries are deprived of healthcare access due to catastrophic out-of-pocket expenditures [46]. Overall, strengthening and scaling up alternative means of supporting these marginalized groups, such as *‘maternity waiting home’* programs [47–49], could reduce out-of-pocket expenditures and increase the EmONC service utilization.

Another important implication is that the CBHI, introduced in 2011in Ethiopia [31], had an evident effect on the health-seeking behavior of society in general and supplying facilities with medical infrastructure. Notably, the service fee exemption [50] was reported to positively impact the uptake of EmONC services in the study area. However, ensuring the application of policies into practice is required since many facilities were facing shortages of medical supplies (though they are supposed to give free of charge). Nevertheless, if further efforts are exerted, including community awareness and testing and applying new innovations that are tried elsewhere [51–53], the improvement of maternal and newborn health is not too far to achieve.

### Health facility-related factors

The existence of basic and comprehensive EmONC service training, Integrated Management of Newborn and Childhood Illness (IMNCI) training, and others were mentioned as contributors to women’s EmONC service use. The evidence stated that a well-performing workforce is needed to achieve the best health outcome [24]. Hence, in-service training had a crucial role in enhancing competency and reducing unnecessary referrals. This ultimately attracts women who perceive poor care provider skills in the facilities [54].

Long waiting time was also identified as a barrier to EmONC service utilization after arrival in the facility. The third delay resulted in worsening complications and contributed to patients’ dissatisfaction and failure to visit EmONC facilities. Therefore, Interventions tried elsewhere, which can address the long waiting time, including obstetric triage improvement programs, can be tested and applied to decrease the waiting time, enhance patients’ satisfaction, and increase service uptake [55].

The lack of wise utilization of resources, inadequate monitoring and evaluation, and poor leadership were identified as barriers to EmONC service utilization in the study area. It was raised that most challenges could have been resolved or lessened with good leadership. For instance, the irrational dispensing/prescription of available supplies, disrespect and abuse, and inequitable distribution of logistics could have been resolved with good leadership. Furthermore, exploring other available sources for medical logistics can enhance the quality of care and increase service uptake. Therefore, leadership and governance, as one of the building blocks of the health system [24], need the staff’s and leaders’ commitment to monitor and control the expenditure wisely. It further needs strategies and policy frameworks that ensure the effectiveness of resource utilization, distribution, regulation, and accountability [24].

### Limitations and strengths of the study

The study’s limitation is that women who sought care outside the health facility might have had difficulty expressing their complications since complications need physical examinations and rigorous diagnosis. However, the study involved an adequate number of women with different experiences to unveil thick descriptions to strengthen the trustworthiness of the findings. The study explored EmOC services utilization problems from women’s direct voices and perspectives, which are vital for designing and implementing a contextualized program to address them. Another strength of the study is that data were collected from multiple sources (women with various backgrounds and complications, health care providers, health facility managers, traditional birth attendants, and community leaders). Therefore, the analysis was built by converging data from multiple perspectives to holistically explore the complex situation of EmONC service utilization-related challenges, enhancing the study’s credibility.

## Conclusion and recommendation

Many women and their newborns in the study area suffered severe and life-threatening complications because of non-utilization or delayed utilization of EmONC services. The study identified barriers and enablers of EmONC service utilization. It summarized them under the themes: service users’ perception and experiences, community-related factors, access and availability of services, healthcare financing, and health facility-related factors. A key policy priority should therefore be to plan to enhance women’s awareness, eliminate misconceptions, improve women’s autonomy, and ensure the role of traditional practices on EmONC service utilization. Community awareness programs and interventions to promote birth preparedness and health education can be required to enhance the EmONC service uptake. Researchers and other stakeholders can investigate the extent and effect of community awareness on reducing maternal and newborn morbidity and mortality.

The evidence from this study suggests that service quality improvement has a central role in enabling women to access and use EmONC services. Furthermore, the health systems must emphasize addressing the inequitable distribution of EmONC facilities (geographically) and medical supplies. The care provider training will also play a vital role in eliminating the bottlenecks, including care provider incompetency, unnecessary referrals, and women’s dissatisfaction. Further study can also confirm the outcome of training on improving maternal and newborn morbidity and mortality. Though the CBHI and fee exemption had a crucial role in enabling women, the financial constraints of out-of-pocket expenses need to be addressed to motivate women of poor socioeconomic status. Furthermore, some interventions need intersectoral collaborations beyond the health system, such as strengthening and maintaining a legal framework to control home deliveries, access to essential drugs, training TBAs, and empowering women.

## Supplementary Information

**Additional file 1: **Information sheet, consent to participate in research, and interview guide.

**Additional file 2: **COREQ (COnsolidated criteria for REporting Qualitative research) Checklist.

## Data Availability

The datasets used and/or analyzed during the current study are available from the corresponding author upon reasonable request.

## Abbreviations

CBHI: Community-based Health Insurance
EmONC: Emergency Obstetric and Newborn Care
IDI: Individual In-depth Interviews
KIIs: Key-informant Interviews
LMICs: Low and Middle-income countries
TBA: Traditional Birth Attendant
WHO: World Health Organization
