# Supplementary material for: Barriers and enablers to emergency obstetric and newborn care services use in Wolaita Zone, Southern Ethiopia: a qualitative case study
Source: BMC Public Health. 2022 Nov 16;22:2087. doi: 10.1186/s12889-022-14504-y (PMC9667656; doi:10.1186/s12889-022-14504-y)
Supplement: Supplementary file 1 — Additional file 1: Information sheet, consent to participate in research, and interview guide. [file 12889_2022_14504_MOESM1_ESM.docx]

## Appendix 1: Information sheet and consent to participate in research for women who had obstetric emergencies

- 1. **Information sheet**

Date: ___________________

Greetings, my name is **Mihiretu Alemayehu Arba**. I am a Ph.D. student at the University of KwaZulu-Natal, and former staff of Wolaita Sodo University, my contact address is (+251913213443 or E-mail address: mihiretua@gmail.com). You are being invited to consider participating in a study that involves research in **“Barriers and Enablers to Emergency Obstetric and Newborn Care Services Use in Wolaita Zone, southern Ethiopia: A Qualitative Case Study”**. The aim and purpose of this research are to learn about the barriers and enablers of service utilization. The study is expected to enroll up to 28 women with obstetric complications in the Wolaita zone though it depends on the information we get. It will involve the following procedures. We are going to interview you, record your audio and take notes. The duration of your participation, if you choose to enroll and remain in the study, is expected to be 45 minutes. This study is funded by the University of KwaZulu-Natal.

The study may involve minimum risk or discomfort. By participating in this research project, you may have minimum discomfort to disclose your painful experience with pregnancy and childbirth-related complications. Many women, however, may find it to be helpful to have the opportunity to talk. We (me and the research assistant) will provide counseling at the end of the interview if you get any discomfort. We will also refer and link you to a counseling service in the health facility. There is no direct benefit to your participation, but we hope the study helps to generate evidence to assist with the improvement of Emergency Obstetric and Neonatal Care service provision in the locality and other similar settings.

This study has been ethically reviewed and approved by the UKZN Biomedical Research Ethics Committee (approval number ___________).

In the event of any problems or concerns/questions you may contact the researcher Mr. Mihiretu Alemayehu (+251913213443 or E-mail address: mihiretua@gmail.com) or the UKZN Biomedical Research Ethics Committee, contact details as follows:

**BIOMEDICAL RESEARCH ETHICS ADMINISTRATION**

Research Office, Westville Campus

Govan Mbeki Building

Private Bag X 54001
Durban
4000

KwaZulu-Natal, SOUTH AFRICA

Tel: 27 31 2604769 - Fax: 27 31 2604609

E-mail: [BREC@ukzn.ac.za](mailto:ngwenyap@ukzn.ac.za)

Your participation in this research is entirely voluntary. You may withdraw the participation at any point, and in the event of refusal/withdrawal of participation, you will not incur a penalty or loss of treatment or other benefits to which you usually are entitled. If at any point you would prefer to refuse the interview, please feel free to tell me. We will terminate you from the study as soon as we receive a refusal/withdrawal. You will not be incurred any cost as a result of participation in this study. There is no direct benefit to your participation, but many women have found it helpful to have the opportunity to talk.

I kindly inform you that all the information you are going to give me will be kept secret. The researchers will not keep the record of your name or address in any of the study’s documents. Any information you provide us including your audio record will be stored securely and kept confidential. The audio record will be stored in a password-protected computer. The publications that will arise from this study will exclude any information that exposes your identification.

- 1. **Consent to participate in research**

I __________________________________ have been informed about the study entitled (**Effective Coverage of Emergency Obstetric and Neonatal Care service in Wolaita Zone, Southern Ethiopia: Context, Correlates and Implications**) by (Mr Mihiretu Alemayehu Arba).

I understand the purpose and procedures of the study.

I have been given an opportunity to answer questions about the study and have had answers to my satisfaction.

I declare that my participation in this study is entirely voluntary and that I may withdraw at any time without affecting any treatment or care that I would usually be entitled to.

I have been informed about any available compensation or medical treatment if an injury occurs to me as a result of study-related procedures.

If I have any further questions/concerns or queries related to the study I understand that I may contact the researcher at +251913213443 or E-mail address: mihiretua@gmail.com.

If I have any questions or concerns about my rights as a study participant, or if I am concerned about an aspect of the study or the researchers then I may contact:

**BIOMEDICAL RESEARCH ETHICS ADMINISTRATION**

Research Office, Westville Campus

Govan Mbeki Building

Private Bag X 54001
Durban
4000

KwaZulu-Natal, SOUTH AFRICA

Tel: 27 31 2604769 - Fax: 27 31 2604609

Email: [BREC@ukzn.ac.za](mailto:ngwenyap@ukzn.ac.za)

**____________________ ____________________**

**Signature of Participant Date**

**____________________ _____________________**

**Signature of Witness Date**

**____________________ _____________________**

**Signature of Interviewer Date**

## Individual in-depth interview guide for women who had obstetric emergencies

NB: *This section will be assessed by conducting individual in-depth interviews using a tape-record/note-taking of the responses of women who had at least one obstetric or neonatal emergency namely: hemorrhage (antepartum or postpartum), prolonged or obstructed labor, postpartum sepsis, complications of abortion, severe pre-eclampsia and eclampsia, ectopic pregnancy, ruptured uterus, or neonatal sepsis/asphyxia*.

1. Please tell me what you understand about obstetric emergencies and what you would do if it happens.

Probe

- What is an obstetric emergency, and how is it treated?
- Please tell me your personal experiences and what you did when it happened to you.
- Where did you seek care? Why did you seek care from there?

1. How did you feel about the quality of EmONC services you received in the hospital/health center? (If the woman used EmONC services)

Probe

- How was the quality of the infrastructure of the facility?
- How did you feel about the care provision processes?
- How do you rate your interaction with care providers and other staff (interpersonal quality)?
- How do you see the reception in the health facility (the care providers and other staff)?
- How confident were the care providers in providing the best treatment and care?
- How do you explain the respect that the health care providers have shown you?
- What was your experience of disrespect and abuse in the facility during service use?
- How did you feel about the availability of drugs, supplies, and equipment required for the service?
- How is your satisfaction with the care?
- What do you think is the reason for the good/poor quality of the care?

1. Why did you not use EmONC services in the health facilities? (If the woman did not use the EmONC services)

Probe

- What were the reasons you did not use EmONC services?
- What were the geographic, economic, social, and political factors that made your decision to use or not use the EmONC services?
- What were your personal/family/community-related reasons for not using?
- Tell me if you had any good/bad experiences with health facilities in the past that impacted your decision regarding not using EmONC services

1. What are the challenges you or someone you know faced in EmONC services?

Probe

- What do you think caused them?
- What should have been done by the facility and care providers?
- How should the services be provided?

1. Please tell me your intentions of where you would prefer to give your future delivery?

Probes

- Where will you seek care if you have an obstetric emergency in the future?
- What conditions and quality elements will encourage to use of EmONC services? Why?
- Where will you recommend other women (with similar emergencies) to get quality service?
- Why did you recommend it to them?

1. How does the community perceive the EmONC service provided in your nearest facility?

Probe

- What is/are the main reason/s for doing so?

1. How should the health facility work on delivery care for you to get the best emergency obstetric care?

Probe

- How should the facility operate?
- What areas need improvements and how should they be improved?
- What should the care providers, health facility administration, district/woreda administration, community, or husband/family do?

**Appendix 2: Information sheet and consent to participate in research for EmONC service providers**

- 1. **Information sheet**

Date: ___________________

Greetings, my name is **Mihiretu Alemayehu Arba**. I am a Ph.D. student at the University of KwaZulu-Natal, and former staff of Wolaita Sodo University, my contact address is (+251913213443 or E-mail address: mihiretua@gmail.com). You are being invited to consider participating in a study that involves research in **“Barriers and Enablers to Emergency Obstetric and Newborn Care Services Use in Wolaita Zone, southern Ethiopia: A Qualitative Case Study”**. The aim and purpose of this research are to learn about the barriers and enablers of service utilization. The study is expected to enroll up to 14 care providers in the 14 selected health facilities of the Wolaita zone though it depends on the information we get. It will involve the following procedures. We are going to interview you, record your audio and take notes. The duration of your participation, if you choose to enroll and remain in the study, is expected to be 45 minutes. This study is funded by the University of KwaZulu-Natal.

The study may involve minimum risk or discomfort. By participating in this research project, you may have minimum discomfort to disclose your painful experience with treating women with pregnancy and childbirth-related complications. Many care providers, however, may find it to be helpful to have the opportunity to talk. We (me and the research assistant) will provide counseling at the end of the interview if you get any discomfort. We will also refer and link you to a counseling service in the health facility. There is no direct benefit to your participation, but we hope the study helps to generate evidence to assist with the improvement of Emergency Obstetric and Neonatal Care service provision in the locality and other similar settings.

This study has been ethically reviewed and approved by the UKZN Biomedical Research Ethics Committee (approval number ___________).

In the event of any problems or concerns/questions you may contact the researcher Mr. Mihiretu Alemayehu (+251913213443 or E-mail address: mihiretua@gmail.com) or the UKZN Biomedical Research Ethics Committee, contact details are as follows:

**BIOMEDICAL RESEARCH ETHICS ADMINISTRATION**

Research Office, Westville Campus

Govan Mbeki Building

Private Bag X 54001
Durban
4000

KwaZulu-Natal, SOUTH AFRICA

Tel: 27 31 2604769 - Fax: 27 31 2604609

E-mail: [BREC@ukzn.ac.za](mailto:ngwenyap@ukzn.ac.za)

Your participation in this research is entirely voluntary. You may withdraw the participation at any point, and in the event of refusal/withdrawal of participation, you will not incur a penalty or loss of treatment or other benefits to which you usually are entitled. If at any point you would prefer to refuse the interview, please feel free to tell me. We will terminate you from the study as soon as we receive a refusal/withdrawal. You will not be incurred any cost as a result of participation in this study. There is no direct benefit to your participation, but many car providers have found it helpful to have the opportunity to talk.

I kindly inform you that all the information you are going to give me will be kept secret. The researchers will not keep the record of your name or address in any of the study’s documents. Any information you provide us including your audio record will be stored securely and kept confidential. The audio record will be stored in a password-protected computer until five years. The publications that will arise from this study will exclude any information that exposes your identification.

- 1. **Consent to participate in research**

I __________________________________ have been informed about the study entitled (**Effective Coverage of Emergency Obstetric and Neonatal Care service in Wolaita Zone, Southern Ethiopia: Context, Correlates and Implications**) by (Mr Mihiretu Alemayehu Arba).

I understand the purpose and procedures of the study.

I have been given an opportunity to answer questions about the study and have had answers to my satisfaction.

I declare that my participation in this study is entirely voluntary and that I may withdraw at any time without affecting any treatment or care that I would usually be entitled to.

I have been informed about any available compensation or medical treatment if an injury occurs to me as a result of study-related procedures.

If I have any further questions/concerns or queries related to the study I understand that I may contact the researcher at +251913213443 or E-mail address: mihiretua@gmail.com.

If I have any questions or concerns about my rights as a study participant, or if I am concerned about an aspect of the study or the researchers then I may contact:

**BIOMEDICAL RESEARCH ETHICS ADMINISTRATION**

Research Office, Westville Campus

Govan Mbeki Building

Private Bag X 54001
Durban
4000

KwaZulu-Natal, SOUTH AFRICA

Tel: 27 31 2604769 - Fax: 27 31 2604609

Email: [BREC@ukzn.ac.za](mailto:ngwenyap@ukzn.ac.za)

**____________________ ____________________**

**Signature of Participant Date**

**____________________ _____________________**

**Signature of Witness Date**

**____________________ _____________________**

**Signature of Interviewer Date**

## Key-informant Interview guide for EmONC service providers

NB: *This section will be assessed by conducting individual in-depth interviews using a tape-record/note-taking of the responses of EmONC service providers*.

1. Please tell me what you understand about and your experiences of EmONC services?

Probe

- What is the EmONC service?
- What are your personal experiences as an EmONC service provider?
- To what extent are EmONC services provided in this facility and how standardized are they?
- Who should use the EmONC service?
- Who is using EmONC services in your facility? (Rich/poor/urban/rural/educated/uneducated/employed/unemployed?) Why?
- Where and when is the service provided?

1. How do you explain the quality of EmONC service provided in your facility?

Probe

- What do you think is the reason for the poor/good quality of the facility?
- How qualified are the care providers to provide EmONC services?
- How prepared is your facility to provide quality EmONC (infrastructure, human resources, logistics, operation time, amenities to care, etc.)?
- How do you see the waiting time to obtain EmONC in your facility?
- How do you see the competency of the care providers on EmONC services? Why?
- How do you welcome and approach patients?
- To what extent are the clients respected in your facility? What do you understand about abuse and disrespect?
- How do your colleagues/co-workers respect their patients?
- What happens to care providers who abuse and disrespect maternity care clients in your facility? Give examples
- What are the missing elements that negatively impact the quality of EmONC in your facility?

1. How do your patients/clients perceive receiving quality treatment for obstetric emergencies at your facility?

Probe

- How do you assess if your clients are satisfied with the quality of care you provide in this facility?
- How do you rate their satisfaction with the care your facility provides?
- What do the patients expect from you?
- How do you see the outcomes of EmONC service in your facility (death, cure, or complications)?

1. What conditions should be fulfilled in your facility for you to provide EmONC services?

- Why are these conditions important?
- Who should fulfill them?
- How should the facility operate?
- What should the care providers do? health facility administration do? district/woreda administration do? community does? patients do? husband/family of the patients do?

**Appendix 3: Information sheet and consent to participate in research for kebele and health development army leaders**

- 1. **Information sheet**

Date: ___________________

Greetings, my name is **Mihiretu Alemayehu Arba**. I am a Ph.D. student at the University of KwaZulu-Natal, and former staff of Wolaita Sodo University, my contact address is (+251913213443 or E-mail address: mihiretua@gmail.com). You are being invited to consider participating in a study that involves research in **“Barriers and Enablers to Emergency Obstetric and Newborn Care Services Use in Wolaita Zone, southern Ethiopia: A Qualitative Case Study”**. The aim and purpose of this research are to learn about the barriers and enablers of service utilization. The study is expected to enroll up to 5 kebele and health development army leaders in the catchment areas of the 14 selected health facilities of the Wolaita zone though it depends on the information we get. It will involve the following procedures. We are going to interview you, record your audio and take notes. The duration of your participation, if you choose to enroll and remain in the study, is expected to be 45 minutes. This study is funded by the University of KwaZulu-Natal.

The study may involve minimum risk or discomfort. By participating in this research project, you may have minimum discomfort to disclose your experience with women who had a pregnancy and childbirth-related complications. Many care providers, however, may find it to be helpful to have the opportunity to talk. We (me and the research assistant) will provide counseling at the end of the interview if you get any discomfort. We will also refer and link you to a counseling service in the health facility. There is no direct benefit to your participation, but we hope the study helps to generate evidence to assist with the improvement of Emergency Obstetric and Neonatal Care service provision in the locality and other similar settings.

This study has been ethically reviewed and approved by the UKZN Biomedical Research Ethics Committee (approval number ___________).

In the event of any problems or concerns/questions you may contact the researcher Mr Mihiretu Alemayehu (+251913213443 or E-mail address: mihiretua@gmail.com) or the UKZN Biomedical Research Ethics Committee, contact details as follows:

**BIOMEDICAL RESEARCH ETHICS ADMINISTRATION**

Research Office, Westville Campus

Govan Mbeki Building

Private Bag X 54001
Durban
4000

KwaZulu-Natal, SOUTH AFRICA

Tel: 27 31 2604769 - Fax: 27 31 2604609

E-mail: [BREC@ukzn.ac.za](mailto:ngwenyap@ukzn.ac.za)

Your participation in this research is entirely voluntary. You may withdraw the participation at any point, and in the event of refusal/withdrawal of participation, you will not incur a penalty or loss of treatment or other benefits to which you usually are entitled. If at any point you would prefer to refuse the interview, please feel free to tell me. We will terminate you from the study as soon as we receive a refusal/withdrawal. You will not be incurred any cost as a result of participation in this study. There is no direct benefit to your participation, but many participants have found it helpful to have the opportunity to talk.

I kindly inform you that all the information you are going to give me will be kept secret. The researchers will not keep the record of your name or address in any of the study’s documents. Any information you provide us including your audio record will be stored securely and kept confidential. The audio record will be stored in a password-protected computer until five years. The publications that will arise from this study will exclude any information that exposes your identification.

- 1. **Consent to participate in research**

I __________________________________ have been informed about the study entitled (**Effective Coverage of Emergency Obstetric and Neonatal Care service in Wolaita Zone, Southern Ethiopia: Context, Correlates and Implications**) by (Mr Mihiretu Alemayehu Arba).

I understand the purpose and procedures of the study.

I have been given an opportunity to answer questions about the study and have had answers to my satisfaction.

I declare that my participation in this study is entirely voluntary and that I may withdraw at any time without affecting any treatment or care that I would usually be entitled to.

I have been informed about any available compensation or medical treatment if injury occurs to me as a result of study-related procedures.

If I have any further questions/concerns or queries related to the study I understand that I may contact the researcher at +251913213443 or E-mail address: mihiretua@gmail.com.

If I have any questions or concerns about my rights as a study participant, or if I am concerned about an aspect of the study or the researchers then I may contact:

**BIOMEDICAL RESEARCH ETHICS ADMINISTRATION**

Research Office, Westville Campus

Govan Mbeki Building

Private Bag X 54001
Durban
4000

KwaZulu-Natal, SOUTH AFRICA

Tel: 27 31 2604769 - Fax: 27 31 2604609

Email: [BREC@ukzn.ac.za](mailto:ngwenyap@ukzn.ac.za)

**____________________ ____________________**

**Signature of Participant Date**

**____________________ _____________________**

**Signature of Witness Date**

**____________________ _____________________**

**Signature of Interviewer Date**

## Key Informant Interview guide for kebele and health development army leaders

NB: *This section will be assessed by conducting a key-informant interview using a tape-record/note-taking of the responses of kebele and health development army leaders*.

1. Please tell me what you understand about and your experiences of EmONC services?

Probe

- What is EmONC? What are the conditions that necessitate EmONC?
- Who should use the EmONC service?
- Where and when is the service provided?
- Where should women seek care for obstetric emergencies?
- Why should they seek care from there?
- What should a woman do when she faces obstetric emergencies?

1. How do you explain the geographical accessibility and availability of the nearest EmONC facility? (explain EmONC to the respondent)

Probe

- How do you see the accessibility of transportation services?
- How do you see the travel cost and distance?
- What do you think affects the timely arrival of women with obstetric emergencies to reach the health facility?
- How do you feel about the service cost that women are obligated to pay?
- How do you feel about the availability of drugs, medical equipment, and laboratory services?

1. How do you perceive the quality of EmONC service provided at health facilities in your area?

Probe

- What is the reason for the good/poor quality?
- How do care providers welcome and approach women with obstetric emergencies?
- How is the respect of the facility’s staff for the patients?
- How is their facial expression toward their patients?
- What do you feel about the care the health professional gives to patients?

1. Which health facility do women in your locality prefer to visit when they face obstetric emergencies?

- Why do they (or don’t) prefer the mentioned facility?
- What are the strengths of the mentioned facility?

1. What do you recommend for the improvement of women’s utilization of quality EmONC service?

Probe

- How should the facility operate?
- What areas need improvements and how should they be improved?
- What should the husband/family do? care providers do? health facility, woreda, or zonal health administration do?

## Appendix 4: Information sheet and consent to participate in research for traditional birth attendants

- 1. **Information sheet**

Date: ___________________

Greetings, my name is **Mihiretu Alemayehu Arba**. I am a Ph.D. student at the University of KwaZulu-Natal, and former staff of Wolaita Sodo University, my contact address is (+251913213443 or E-mail address: mihiretua@gmail.com). You are being invited to consider participating in a study that involves research in **“Barriers and Enablers to Emergency Obstetric and Newborn Care Services Use in Wolaita Zone, southern Ethiopia: A Qualitative Case Study”**. The aim and purpose of this research are to learn about how women with pregnancy and childbirth-related complications get quality service and what factors affect the use of quality service. The study is expected to enroll up to 5 traditional birth attendants in the Wolaita zone though it depends on the information we get. It will involve the following procedures. We are going to interview you, record your audio and take notes. The duration of your participation, if you choose to enroll and remain in the study, is expected to be 45 minutes. This study is funded by the University of KwaZulu-Natal.

The study may involve minimum risk or discomfort. By participating in this research project, you may have minimum discomfort to disclose your experience with women who had pregnancy and childbirth-related complications. Many care providers, however, may find it to be helpful to have the opportunity to talk. We (me and the research assistant) will provide counseling at the end of the interview if you get any discomfort. We will also refer and link you to a counseling service in the health facility. There is no direct benefit to your participation, but we hope the study helps to generate evidence to assist with the improvement of Emergency Obstetric and Neonatal Care service provision in the locality and other similar settings.

This study has been ethically reviewed and approved by the UKZN Biomedical Research Ethics Committee (approval number ___________).

In the event of any problems or concerns/questions you may contact the researcher Mr Mihiretu Alemayehu (+251913213443 or E-mail address: mihiretua@gmail.com) or the UKZN Biomedical Research Ethics Committee, contact details as follows:

**BIOMEDICAL RESEARCH ETHICS ADMINISTRATION**

Research Office, Westville Campus

Govan Mbeki Building

Private Bag X 54001
Durban
4000

KwaZulu-Natal, SOUTH AFRICA

Tel: 27 31 2604769 - Fax: 27 31 2604609

E-mail: [BREC@ukzn.ac.za](mailto:ngwenyap@ukzn.ac.za)

Your participation in this research is entirely voluntary. You may withdraw the participation at any point, and in the event of refusal/withdrawal of participation, you will not incur a penalty or loss of treatment or other benefits to which you usually are entitled. If at any point you would prefer to refuse the interview, please feel free to tell me. We will terminate you from the study as soon as we receive a refusal/withdrawal. You will not be incurred any cost as a result of participation in this study. There is no direct benefit to your participation, but many participants have found it helpful to have the opportunity to talk.

I kindly inform you that all the information you are going to give me will be kept secret. The researchers will not keep the record of your name or address in any of the study’s documents. Any information you provide us including your audio record will be stored securely and kept confidential. The audio record will be stored in a password-protected computer until five years. The publications that will arise from this study will exclude any information that exposes your identification.

- 1. **Consent to participate in research**

I __________________________________ have been informed about the study entitled (**Effective Coverage of Emergency Obstetric and Neonatal Care service in Wolaita Zone, Southern Ethiopia: Context, Correlates and Implications**) by (Mr Mihiretu Alemayehu Arba).

I understand the purpose and procedures of the study.

I have been given an opportunity to answer questions about the study and have had answers to my satisfaction.

I declare that my participation in this study is entirely voluntary and that I may withdraw at any time without affecting any treatment or care that I would usually be entitled to.

I have been informed about any available compensation or medical treatment if an injury occurs to me as a result of study-related procedures.

If I have any further questions/concerns or queries related to the study I understand that I may contact the researcher at +251913213443 or E-mail address: mihiretua@gmail.com.

If I have any questions or concerns about my rights as a study participant, or if I am concerned about an aspect of the study or the researchers then I may contact:

**BIOMEDICAL RESEARCH ETHICS ADMINISTRATION**

Research Office, Westville Campus

Govan Mbeki Building

Private Bag X 54001
Durban
4000

KwaZulu-Natal, SOUTH AFRICA

Tel: 27 31 2604769 - Fax: 27 31 2604609

Email: [BREC@ukzn.ac.za](mailto:ngwenyap@ukzn.ac.za)

**____________________ ____________________**

**Signature of Participant Date**

**____________________ _____________________**

**Signature of Witness Date**

**____________________ _____________________**

**Signature of Interviewer Date**

- 1. **Key Informant Interviews guide for traditional birth attendants**

NB: *This section will be assessed by conducting a key-informant interview using a tape-record/note-taking of the responses of traditional birth attendants*.

1. Please tell me what you understand about and your experiences of EmONC services?

Probe

- What is EmONC? What are the conditions that necessitate EmONC?
- Who should use the EmONC service?
- Where and when is the service provided?
- Where should women seek care for obstetric emergencies?
- Why should they seek care from there?
- What should a woman do when she faces obstetric emergencies?

1. What do you do when a woman with an obstetric emergency comes to you?

Probe

- How do you take care of her?
- How do you manage the obstetric problem the patient is suffering?
- What do you do when the obstetric emergency is difficult to be managed by you?
- Why do you do it?

1. How do you feel about the quality of obstetric care you provide to women with emergencies?

Probe

- What is the reason for the good/poor quality of the service you provide?
- How do you welcome and respect her?
- How is your facial expression to the patient?
- What do you think patients need from you?

1. How do you feel about the quality of EmONC service provided at health facilities?

Probe

- How do you feel about the EmONC service quality in the government health facilities in general?
- How do you feel about the quality at the nearest health facility in your locality?
- What do you think is the reason for the good/poor quality of the facility?
- How do you see the quality of obstetric care given at government facilities as compared to the service given by traditional birth attendants?
- Why do women prefer visiting traditional birth attendants (including you) than health facilities for pregnancy emergencies?
- What makes your service better than that of government facilities?
- Why do you think the health facilities can’t provide the mentioned service?
- What do you think the health facilities can provide that you can’t provide?

1. What do you recommend for the improvement of women’s utilization of EmONC service?

Probe

- Who should do what?
- What should women do? traditional birth attendants do? husband/family do? care providers of health facilities do? health facility administration do? district/woreda administration do?

## Appendix 5: Information sheet and consent to participate in research for health facility, district, and zonal health office managers

- 1. **Information sheet**

Date: ___________________

Greetings, my name is **Mihiretu Alemayehu Arba**. I am a Ph.D. student at the University of KwaZulu-Natal, and former staff of Wolaita Sodo University, my contact address is (+251913213443 or E-mail address: mihiretua@gmail.com). You are being invited to consider participating in a study that involves research in **“Barriers and Enablers to Emergency Obstetric and Newborn Care Services Use in Wolaita Zone, southern Ethiopia: A Qualitative Case Study”**. The aim and purpose of this research are to learn about how women with pregnancy and childbirth-related complications get quality service and what factors affect the use of quality service. The study is expected to enroll up to 8 health facility managers in the Wolaita zone though it depends on the information we get. It will involve the following procedures. We are going to interview you, record your audio and take notes. The duration of your participation, if you choose to enroll and remain in the study, is expected to be 45 minutes. The University of KwaZulu-Natal funds this study.

The study may involve minimum risk or discomfort. By participating in this research project, you may have minimum discomfort to disclose your painful experience helping women with pregnancy and childbirth-related complications. Many facility managers, however, may find it to be helpful to have the opportunity to talk. We (me and the research assistant) will provide counseling at the end of the interview if you get any discomfort. We will also refer and link you to a counseling service in the health facility. There is no direct benefit to your participation, but we hope the study helps to generate evidence to assist with the improvement of Emergency Obstetric and Neonatal Care service provision in the locality and other similar settings.

This study has been ethically reviewed and approved by the UKZN Biomedical Research Ethics Committee (approval number ___________).

In the event of any problems or concerns/questions you may contact the researcher Mr Mihiretu Alemayehu (+251913213443 or E-mail address: mihiretua@gmail.com) or the UKZN Biomedical Research Ethics Committee, contact details as follows:

**BIOMEDICAL RESEARCH ETHICS ADMINISTRATION**

Research Office, Westville Campus

Govan Mbeki Building

Private Bag X 54001
Durban
4000

KwaZulu-Natal, SOUTH AFRICA

Tel: 27 31 2604769 - Fax: 27 31 2604609

E-mail: [BREC@ukzn.ac.za](mailto:ngwenyap@ukzn.ac.za)

Your participation in this research is entirely voluntary. You may withdraw the participation at any point, and in the event of refusal/withdrawal of participation, you will not incur a penalty or loss of treatment or other benefits to which you usually are entitled. If at any point you would prefer to refuse the interview, please feel free to tell me. We will terminate you from the study as soon as we receive a refusal/withdrawal. You will not be incurred any cost as a result of participation in this study. There is no direct benefit to your participation, but many participants have found it helpful to have the opportunity to talk.

I kindly inform you that all the information you are going to give me will be kept secret. The researchers will not keep the record of your name or address in any of the study’s documents. Any information you provide us including your audio record will be stored securely and kept confidential. The audio record will be stored in a password-protected computer until five years. The publications that will arise from this study will exclude any information that exposes your identification.

- 1. **Consent to participate in research**

I __________________________________ have been informed about the study entitled (**Effective Coverage of Emergency Obstetric and Neonatal Care service in Wolaita Zone, Southern Ethiopia: Context, Correlates and Implications**) by (Mr Mihiretu Alemayehu Arba).

I understand the purpose and procedures of the study.

I have been given an opportunity to answer questions about the study and have had answers to my satisfaction.

I declare that my participation in this study is entirely voluntary and that I may withdraw at any time without affecting any treatment or care that I would usually be entitled to.

I have been informed about any available compensation or medical treatment if an injury occurs to me as a result of study-related procedures.

If I have any further questions/concerns or queries related to the study I understand that I may contact the researcher at +251913213443 or E-mail address: mihiretua@gmail.com.

If I have any questions or concerns about my rights as a study participant, or if I am concerned about an aspect of the study or the researchers then I may contact:

**BIOMEDICAL RESEARCH ETHICS ADMINISTRATION**

Research Office, Westville Campus

Govan Mbeki Building

Private Bag X 54001
Durban
4000

KwaZulu-Natal, SOUTH AFRICA

Tel: 27 31 2604769 - Fax: 27 31 2604609

Email: [BREC@ukzn.ac.za](mailto:ngwenyap@ukzn.ac.za)

**____________________ ____________________**

**Signature of Participant Date**

**____________________ _____________________**

**Signature of Witness Date**

**____________________ _____________________**

**Signature of Interviewer Date**

## Key informant interview guide for the health center, hospital, district, and zonal health office managers)

NB: *This section will be assessed by conducting key-informant interviews using a tape-record/note-taking of the responses of health facility managers*.

1. Please tell me what you understand about and your programmatic experiences of EmONC services?

Probe

- What do you understand about EmONC?
- Who should use the EmONC service?
- What is your overall assessment of EmONC services in your area (access, quality, affordability, acceptability, coverage, etc.)?
- Where and when is the service provided in your area?
- Who is using and who is not using EmONC services in your area? (Rich/poor/urban/rural/educated/uneducated/employed/unemployed?) Why?

1. How do you explain the quality of EmONC service provided in the health facility/facilities under your supervision?

Probe

- What do you think is the reason for the poor/good quality of the facility?
- How qualified are the care providers to provide EmONC services?
- How prepared is your facility/woreda/zone to provide quality EmONC (infrastructure, human resources, logistics, operation time, amenities to care, etc.)?
- How do you see the waiting time to obtain EmONC in your facility?
- How do see the competency of the care providers on EmONC services in your facility/woreda/zone? Why?
- To what extent are the EmONC clients respected in your facility/woreda/zone?
- What do you understand about abuse and disrespect?
- How is disrespect and abuse of EmONC clients managed in your facility/woreda/zone?
- What happens to care providers who abuse and disrespect maternity care clients in your facility/woreda/zone? What mechanism is in place? Give examples
- How do you assess if EmONC clients are satisfied with the quality of care you provide in this facility/woreda/zone? How do you assess or obtain the patients’ voice of quality of care?
- How do you see the outcomes of EmONC service in your facility (death, cure, or complications)?
- What are the missing elements that negatively impact the quality of EmONC in your facility/woreda/zone?

1. How do patients with obstetric emergencies perceive the quality of EmONC service provided in your facility/facilities?

Probe

- How do you rate their satisfaction with the care your facility provides?
- What do the patients expect from the facility/facilities?

1. Which facility/facilities provide a good quality EmONC service?

- How did you select them for the good quality service?
- What strength do the facilities have?
- Why are the other facilities categorized as relatively poor-quality service providers?
- What is the reason for the poor quality?

1. What do you recommend for the improvement of the quality of EmONC services?

Probe

- How should the facilities operate?
- What areas need improvements for the improvement of care?
- How should they be improved?
- How should the guidelines/protocols be prepared?
- What should the care providers do?

health facility administration do?

district/woreda administration do?

community does?

patients do?

husband/family of the patients do?
